# Sleep Education Program with Self-Help Treatment—Sleep-Promoting Behaviors for Children and Adolescents in Japan

**DOI:** 10.3390/children13010092

**Published:** 2026-01-08

**Authors:** Hideki Tanaka, Norihisa Tamura, Kaori Yamaoka, Taro Matsuki

**Affiliations:** 1 Department of Psychology, Faculty of Health and Wellness Sciences, Hiroshima International University, 555-36, Kurose-Gakuendai, Higashihiroshima 739-2695, Hiroshima, Japan; kaimin.suimin.ouen@gmail.com (K.Y.); t-matsuki@hirokoku-u.ac.jp (T.M.); 2 Department of Psychology, Graduate School of Humanities & Social Sciences, Hiroshima University, 1-1-1 Kagamiyama, Higashihiroshima 739-8524, Hiroshima, Japan; tamura65@hiroshima-u.ac.jp

**Keywords:** adolescents, children, insufficient sleep, self-help treatment, sleep education

## Abstract

Late bedtimes and insufficient sleep duration among children and adolescents have been consistently associated with daytime sleepiness, irritability, and poorer academic performance. To mitigate these adverse consequences of insufficient sleep, it is essential to provide children, students, teachers, and parents with not only knowledge about sleep improvement but also practical tools that facilitate behavioral change. This review synthesizes existing evidence from studies that have addressed this issue by evaluating students’ individual sleep behaviors using checklists of sleep-promoting practices. Drawing on practical examples from school-based interventions, the review highlights the effectiveness of sleep education programs for children and adolescents. These programs aim to bridge the gap between sleep-related knowledge and actual behavioral change by targeting daily sleep habits. Typically, such programs consist of a single 50 min educational session focusing on the importance of sleep and strategies for improvement, followed by a two-week self-help period during which students actively practice and monitor specific target behaviors. Overall, the findings indicate that sleep education programs incorporating self-help components not only enhance sleep-related knowledge but also promote healthier sleep behaviors and improve sleep patterns. Moreover, these programs effectively reduce daytime sleepiness and irritability among children and adolescents, thereby contributing to a healthier and more adaptive school life.

## 1. Introduction

The negative effects of delayed bedtimes in childhood and adolescence—specifically (1) sleep deprivation and (2) disruption of circadian rhythms—are serious from the perspectives of healthy development and both brain and physical/mental health. Japan’s average sleep duration is 7 h and 22 min (aged 15–64), the shortest among 33 OECD countries (including candidate countries) [1]. This trend extends to children exhibiting delayed bedtimes and insufficient sleep. Inadequate sleep in children and adolescents is a major international concern. A review on adolescent sleep specifically notes that, compared to Western countries, Asia exhibits later bedtimes, shorter sleep duration, and stronger daytime sleepiness [2]. A survey conducted by Japan’s Ministry of Education, Culture, Sports, Science and Technology (MEXT) reported that while 1.6% of elementary school students go to bed after midnight, this proportion increases to 22.0% among junior high school students and reaches 47.0% among high school students [3]. Japanese children’s sleep duration falls below the recommended sleep time for their developmental age [4] across all age groups, with approximately one in four junior high school students and one in three high school students experiencing sleep deprivation [3]. Addressing sleep problems in children and adolescents in a realistic and effective manner involves providing sleep education not only to the individuals themselves but also to teachers and parents.

Insufficient or poor sleep is known to impair emotional regulation functions associated with frontal lobe activity, reduce motivation, and increase the risk of impulsivity, anxiety/depressive mood, and obesity [5,6]. It is also linked to impairments in memory and learning, reduced insight, poorer academic performance, weakened immune function and obesity [7,8,9].

Compared to childhood, adolescents are more likely to have a greater discrepancy between the sleep–wake rhythm on school nights and weekends [2,10], which may result in a phase shift of the biological clock and an increased tendency for daytime sleepiness [11,12,13]. Consequently, synchronizing the circadian rhythm with school schedules may be difficult, potentially posing a greater risk of reduced daytime functioning.

According to a cross-sectional study of more than 4000 students aged 12–15 years in 13 junior high schools in Japan, a discrepancy of two hours or more in wake-up time between school days and weekends predicted difficulty initiating sleep (odds ratio [OR] 1.50), poor sleep quality (OR 1.57), insufficient sleep (OR 1.99), daytime sleepiness (OR 2.15), fatigue (OR 1.31), irritability (OR 1.46), and poor academic performance (OR 1.29–1.36) in multivariate analyses [14]. Therefore, sleep education for adolescents should also focus on reducing discrepancies between sleep–wake patterns on school nights and weekends [14,15].

Behavioral and educational interventions are effective approaches for promoting healthy sleep. A combination of sleep education and behavioral strategies has been shown to alleviate sleep problems [16]. These intervention strategies are often provided as individualized support in clinical settings; however, there remains a need to further examine their implementation as school-based, group-oriented interventions for non-clinical populations.

This review aims to provide an overview of the effects of sleep education combined with self-help treatment, a behavioral strategy, for Japanese children and adolescents, drawing on practical examples from school settings.

## 2. Global Trends in Sleep Education

Sleep education programs for children and adolescents have been implemented in various countries, confirming improvements in sleep knowledge [17,18,19,20,21,22,23]. Such programs include education on the importance of sleep, appropriate sleep health, and methods to help modify sleep patterns [24]. While sleep education programs have been shown to be effective in increasing children’s and students’ knowledge about sleep, consistent results with respect to improvements in sleep-related outcomes such as daytime sleepiness, sleep duration, and sleep hygiene behaviors have not been consistently demonstrated [24,25].

Although school-based sleep education has been widely implemented internationally, its effectiveness remains inconsistent. A review analyzing 21 studies involving children and adolescents aged 5–18 years [26] reported that many school-based interventions were limited to short-term, knowledge-based programs (median duration: 4 weeks; median total instructional time: approximately 200 min). As a result, although sleep knowledge improved, the interventions did not lead to clear or consistent improvements in sleep habits (e.g., sleep duration, bedtime) or sleep-related behaviors. The review also pointed out that sleep education alone often lacks key components necessary for behavioral change and is associated with a high risk of bias.

In addition, a study that implemented a four-month sleep education program in rural China [27] reported no significant differences between the intervention and control groups in sleep knowledge, attitudes toward sleep, or sleep quality. Furthermore, a study conducted among high school students in a regional city in Japan [28] found that sleep education had some preventive effects on insomnia symptoms but failed to improve sleep quality, daytime sleepiness, or delayed bedtimes.

On the other hand, a study focusing on the methods of implementing sleep education [29] co-developed a six-session (30 min per session) curriculum that high school teachers could deliver during class time. Although the curriculum demonstrated high feasibility and acceptability, the study also revealed that it remained difficult to improve students’ actual sleep insufficiency (i.e., short sleep duration). In another study targeting military personnel [30], a single 30 min intervention focusing on circadian rhythms, light exposure, and sleep skills significantly improved sleep hygiene behaviors and psychological health, highlighting the importance of incorporating concrete behavioral skills alongside knowledge-based education.

Moreover, in the Japanese context, it has been pointed out that sleep deprivation among children and adolescents is reinforced by sociocultural values such as self-sacrifice, diligence, and asceticism [31], suggesting that classroom-based sleep education alone is insufficient to promote sustained behavioral change.

Taken together, these findings indicate that although traditional school-based sleep education contributes to improvements in sleep knowledge, it has limited effects on changes in sleep habits or sleep hygiene behaviors. Therefore, strategies that promote behavioral change—such as self-monitoring, individualized goal setting, and the integration of essential sleep knowledge with actionable behaviors—are critically important.

### The Importance of Goal Setting

We have developed a checklist for sleep-promoting behaviors and a method for assessing each individual’s implementation status. This approach facilitates the evaluation of each student’s practice of sleep-promoting behaviors and the establishment of achievable goals. It is crucial to provide both knowledge-based sleep education and practical tools that support behavioral change to children, students, teachers, and parents.

## 3. Introducing Sleep Education in Schools

Introducing sleep education in schools requires (1) disseminating accurate knowledge about sleep and circadian rhythms, and (2) helping teachers and parents understand the links between observable problem behaviors (such as dozing off in class or skipping breakfast) and physical and mental health, academic performance, and benefits within school life. Furthermore, when conducting sleep education, it is vital to first help students understand (1) the importance of sleep improvement and (2) key knowledge for improving sleep, thereby encouraging them to reflect on their own lifestyles. It is also crucial to link this essential knowledge about sleep improvement to specific actions, helping students incorporate what they learn into their daily lives as habits. When communicating the importance of sleep improvement, it is vital not only to explain the role of sleep itself but also to provide concrete examples of how improvements in sleep and lifestyle habits can positively transform students’ school life.

### 3.1. Linking Key Sleep Improvement Knowledge to Action

Applying key sleep improvement knowledge to actual behavior and incorporating it into habitual practice is crucial for enhancing sleep quality and daytime functioning. When specific sleep-promoting habits (hereafter, sleep-promoting behaviors) are proposed alongside lectures, understanding is enhanced and behavioral change is more readily facilitated. To achieve this, in addition to disseminating knowledge about sleep and circadian rhythms, tools that can be utilized in student guidance are essential. In our school-based sleep education, we first explain concepts using true/false quizzes about sleep, then guide students to conduct self-assessments of their daily rhythms and set personal goals [16]. This approach effectively links essential sleep knowledge with action. To sustain this practice in schools, opportunities for sleep education for teachers and caregivers must also be created, requiring the establishment of a community-wide support system.

### 3.2. The Importance of Materials Linking Knowledge and Habits

Regarding knowledge to promote sleep improvement, it is crucial to first convey that the conditions for restful sleep include (1) the brain not being aroused and (2) body temperature decreasing smoothly [32]. Possessing this knowledge enables children to independently judge whether a given action is beneficial for restful sleep or disruptive to it.

During lectures and sleep classes (approximately 50 min), explanations incorporate true/false quizzes about sleep, and the “Lifestyle Rhythm Healthy Method” (Sleep-Promoting Behaviors Checklist) is introduced [32]. This connects essential sleep improvement knowledge with specific actions.

① Is a drop in body temperature important for falling asleep easily? (True)
② Is it better not to open the curtains immediately upon waking? (False)
③ Is it good to sleep until the afternoon on days off? (False)
④ If you feel sleepy in the evening after returning home, is it better to take a nap? (False)
⑤ Before bedtime, is it better to avoid bright places like convenience stores? (False)

The latest version of the educational pamphlet can be downloaded from the Hiroshima International University “Min-iku” website [33].

The following explanations are provided for the Quiz (Question).

### 3.3. Cool Head, Warm Feet: Key to Restful Sleep (Explanation of Question *①*)

The key to improving sleep lies first in understanding the relationship between sleep and body temperature rhythms. People fall asleep as body temperature decreases and wake up as it increases. Therefore, a smooth decline in body temperature is essential for restful sleep. When newborns and infants become sleepy, their hands and feet warm up as heat is released from the extremities to lower core body temperature. This “cool head, warm feet” principle is fundamental to restful sleep. Cooling the head and neck during hot summers and warming the feet with a hot water bottle in cold winters is therefore physiologically reasonable. “Cool head” also indicates that the brain is not excessively stimulated.

### 3.4. Tips for Regulating Rhythm (Explanation of Question *②*, *③*, *④*)

To regulate circadian rhythm, it is important to eat breakfast in a bright environment. Because light intake through the eyes plays a critical role, facing the light while eating is recommended. Sleeping late on weekends to compensate for weekday sleep deprivation can disrupt sleep rhythm, leading to difficulty falling asleep at night and feelings of grogginess and discomfort on Monday mornings. Even on weekends, students should aim to wake up at approximately the same time as on weekdays (or keep the difference within 2 h).

Eating breakfast in a bright area where sunlight enters (within 1 m of a window) while chewing thoroughly is recommended. If students feel sleepy during the day, a short nap is the most effective compensatory strategy, provided it does not disrupt circadian rhythm. On the other hand, long afternoon naps or evening dozing not only encourage later bedtimes but also reduce sleep depth. To prevent evening drowsiness, taking a brief nap of 10 min or less before 3 PM is recommended.

### 3.5. Discuss Actions That Are Good or Bad for Sleep (Includes Explanations for *①*, *⑤*)

In simple terms, the conditions for good sleep are (1) the brain is not aroused and (2) body temperature decreases smoothly. Keeping these two principles in mind allows students to evaluate whether specific actions are beneficial or detrimental to sleep.

Intense exercise or hot baths immediately before bedtime excessively raise body temperature, making it harder to fall asleep; a lukewarm bath is preferable. Cognitive activities such as worrying or playing games before bedtime stimulate the brain. Moreover, light emitted from smartphones and tablets contains a high proportion of blue light, which suppresses melatonin secretion, a hormone essential for sleep. Therefore, reducing smartphone and computer use during the hour before bedtime, switching to a blue-light reduction mode, or postponing use until after waking is recommended. It is also effective for students to discuss hypothetical scenarios incorporating these principles.

## 4. The Tool Linking Knowledge to Sleep-Promoting Behaviors

### 4.1. Utilizing the Daily Rhythm Checklist

It is critically important to translate the knowledge gained through sleep education into actual improvements in sleep-promoting behaviors. The authors propose a method referred to as the Lifestyle Rhythm Health Method to address this challenge by creating a checklist of sleep-promoting behaviors. This method is useful because it systematically assesses sleep-promoting behaviors and facilitates the setting of action goals tailored to each individual’s current practice level [16]. Active engagement in the goal-setting process itself increases the likelihood of achieving those goals.

### 4.2. Lifestyle Rhythm Healthy Method (Sleep-Promoting Behaviors Checklist)

Table 1 shows the junior high school version of the “Lifestyle Rhythm Healthy Method” [16], an action-oriented tool for behavior change that translates key knowledge for sleep improvement into concrete actions and helps students set improvement goals. Utilizing the Lifestyle Rhythm Healthy Method Checklist (Table 1), which lists sleep-promoting behaviors aligned with sleep improvement education content, and a sleep diary is also effective for monitoring changes in one’s own lifestyle rhythm and sleep status. Therefore, we provide a detailed explanation of how to use the checklist.

First, have the student mark items they already do with a 〇, items they don’t do but feel they can work towards with a △, and items they feel they cannot manage with an ×. Then, select one goal from the (△) items. Aiming to change an × to an 〇 is too ambitious and often leads to early dropout, so choose an achievable (△) item as the goal. For elementary and junior high school students, setting a single goal is optimal [16]. Improving even one (△) item provides a starting point to interrupt the vicious cycle. Improving one habit often leads to improvements in other related behaviors.

If there are no (△) items, select a goal from the (〇) items and encourage the student to try maintaining it for two weeks to achieve a (◎). It is crucial to praise even minor behavioral changes as successes and encourage their continuation. Translating knowledge into action and habit is the first step toward improving sleep.

## 5. Practical Methods and Packages for Sleep Education

### Practical Methods for Sleep Education

Sleep education packages that can be implemented in approximately 50 min during school classes or health lectures play a crucial role in promoting healthy sleep behaviors [16]. The authors have implemented a sleep education program with self-help treatment at the class level in elementary schools [34] and junior high schools [35].

Here, we describe a structured sleep education package designed for implementation within regular class time. The education package consists of classroom-based sleep knowledge education (sleep true/false question) and the practice and self-monitoring of target behaviors over a two-week period [34,35].

**Figure 1:**
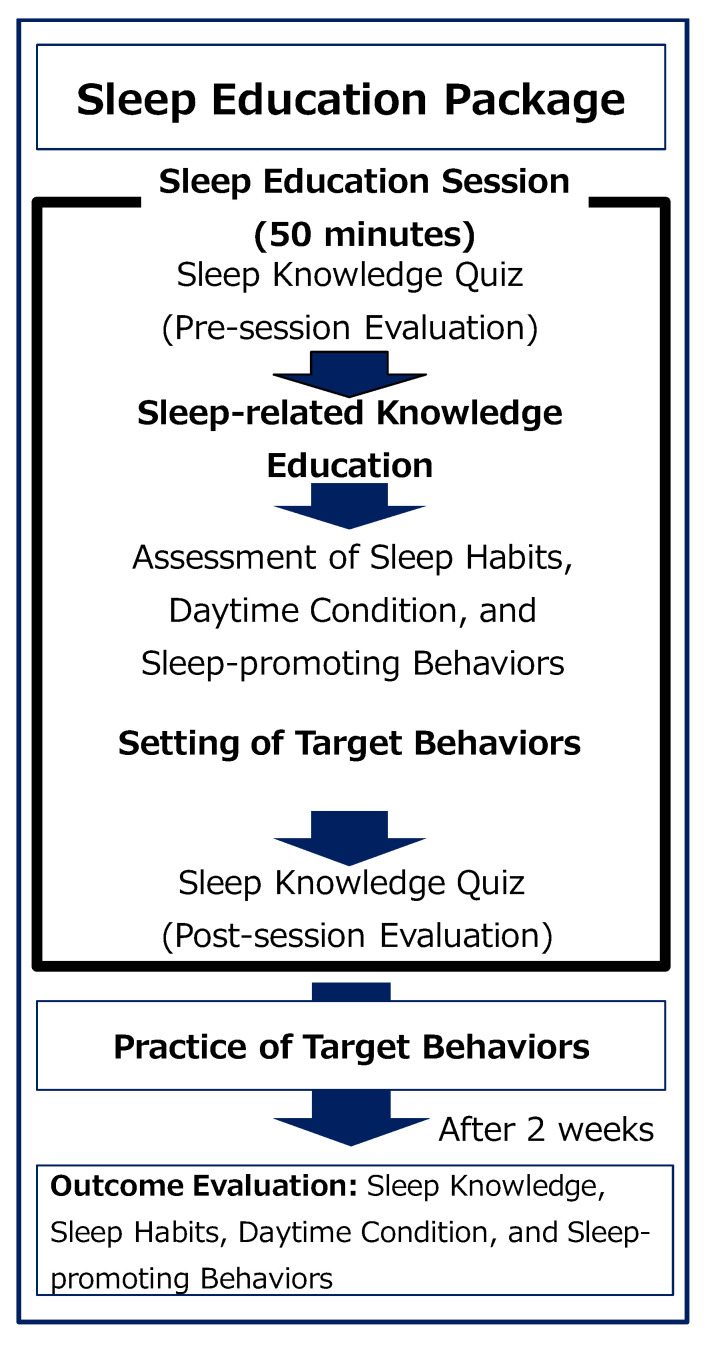
Sleep education package.

Figure 1 shows the overall structure of the 50 min class and the sleep education package. The class flow is as follows: (1) conduct a lecture on sleep incorporating knowledge through a true/false quiz (20 min), (2) assess sleep, daytime states, and lifestyle habits (10 min), (3) provide instruction on sleep log completion (10 min), and (4) confirm knowledge acquisition via a post-class true/false quiz.

Detailed procedures and instructional tools are described below. In the sleep lesson, students first complete a pre-lesson sleep knowledge quiz designed to assess baseline knowledge. Immediately thereafter, the knowledge-based education component begins. During this phase, standardized teaching materials are used to explain the importance of sleep and key concepts for sleep improvement, with feedback provided by reviewing the quiz answers, over approximately 20 min.

Next, using a questionnaire, sleep habits, daytime states, and sleep-promoting behaviors are assessed for approximately 15 min. At this stage, students are encouraged to set achievable goals from among the sleep-promoting behaviors. After the assessment, the procedure for completing the sleep diary is explained for approximately 10 min. Finally, to evaluate the effectiveness of the knowledge education, students complete a post-class sleep knowledge quiz.

Although the classroom-based knowledge education ends at this point, students are instructed to use the sleep log (Appendix A) for approximately two weeks to self-monitor their implementation of the target behaviors and to record bedtime and wake-up times. After the two-week period, effectiveness is evaluated using a similar questionnaire.

This sleep education package implements a series of integrated procedures, including knowledge education (importance of sleep and improvement methods) and lifestyle rhythm healthy methods (lifestyle rhythm checks, goal setting, sleep log recording, and monitoring goal achievement). After two weeks, the following improvements have been reported: (1) increased knowledge about sleep improvement, (2) earlier bedtimes and shorter sleep latency, (3) reduced differences between weekday and weekend bedtimes, and (4) improved mood upon waking and reduced daytime sleepiness [16,34,35]. Furthermore, it is important to adapt the sleep education package according to the developmental stage of children and adolescents. For lower elementary school students, promoting sleep knowledge education and the practice of target behaviors alone is effective in improving sleep duration and daytime sleepiness. In contrast, for upper elementary, junior high, and high school students, combining knowledge education with self-monitoring of target behaviors is expected to be effective in improving sleep duration, bedtime, and irritability.

In addition, recommended sleep-promoting behaviors with high adoption and maintenance rates include: “Get plenty of sunlight upon waking in the morning,” “Avoid napping after returning home,” and “Keep weekend wake-up times within two hours of weekday wake-up times.” When students are observed consciously practicing their goals, praising their efforts and asking, “What did you notice as a result of practicing your goal?” facilitates evaluation of whether goal practice is yielding benefits.

These programs emphasize linking sleep-related knowledge with concrete actions and habits. They consist of a single 50 min session providing knowledge about the importance of sleep and sleep improvement, followed by a two-week self-help treatment focusing on practicing and monitoring target behaviors. This approach has already been implemented and shown to be effective among elementary, junior high, and high school students, school nurses, and community residents [16,34,35,36]. Below, we introduce methods and studies that addressed this issue by evaluating students’ individual sleep behaviors using checklists that include sleep-promoting actions. Based on practical examples in schools, we provide an overview of the effectiveness of sleep education for children and adolescents.

The protocol of the current study was reviewed and approved by the Research Ethics Committee of Hiroshima International University (approval number 12-041). The investigations were conducted in accordance with the principles outlined in the Declaration of Helsinki (1975), and parental consent and student assent, together with consent from school principals and class teachers, were obtained.

## 6. Which Sleep-Promoting Behaviors Are Most Likely to Be Selected?

In instructional settings catering to diverse students, it is also important to understand which sleep-promoting behaviors are most likely to be selected as intervention targets. Sleep education for high school students has also been noted to be effective in improving weekday sleep duration, bedtime, and accumulated sleep debt. Here, to clarify the sleep-promoting behaviors that are crucial for improving high school students’ sleep, we introduce a study that estimated the relationship between target behavior improvement and sleep problem improvement using posterior probabilities. For high school students, they were instructed to select and implement up to three target behaviors from among sleep-promoting behaviors. One month after the sleep education, weekday sleep duration, bedtime, and sleep debt showed improvement.

### 6.1. Changes in Targeted Behavior

First, when examining the selection status of the three goals, all students selected at least one goal. Those selecting two goals and those selecting three goals accounted for 85% (407 students) and 73% (350 students) of the total, respectively. Among these, examining the proportion of students showing improvement in at least one goal revealed that 274 of 478 students (57.3%) demonstrated significant improvement in target behaviors during the Post period (Table 2).

Examining improvements by specific goal behaviors revealed that all goal behaviors showed significant improvement from the Pre period to the Post period. Notably, more than 80% of students who set goals such as “Enjoy hobbies or club activities and spend time actively” and “Avoid going out to bright places like convenience stores after 9 PM” showed improvement in these behaviors. Furthermore, among students who set goals such as “Wake up at roughly the same time every morning,” “Avoid irregular bedtimes,” and “Keep weekend wake-up times within 2 h of weekday times,” more than 60% showed improvement in their target behaviors (Table 2).

### 6.2. Probability of Target Behavior Improvement When Sleep Duration, Bedtime, and Sleep Debt Improve

Results of calculating the posterior probability of target behavior improvement when sleep duration (increased by 40 min or more), bedtime advancement (advanced by 30 min or more), and sleep debt (reduced by 60 min or more) improved are shown in Table 3.

The posterior probabilities for “Avoid letting your wake-up time on days off differ by more than 2 h from weekdays” and “Avoid napping after returning home” were high, reaching 77.1%, 84.0%, and 85.7% for the former, and 77.6%, 74.3%, and 87.4% for the latter. For “Wake up at roughly the same time every morning,” the posterior probabilities were 65.3%, 73.8%, and 73.8%, respectively. For “Get plenty of sunlight after waking up in the morning,” they were 58.3%, 56.4%, and 66.7%, respectively. For “Avoiding caffeine such as tea or coffee after dinner” and “Going to bed by midnight,” the probability values exceeded 80% for sleep duration and bedtime, respectively.

These results suggest that the Sleep Education Program with Self-Help Treatment, which emphasizes linking sleep improvement knowledge with sleep-promoting behaviors, is effective in improving weekday sleep duration, bedtime, and sleep debt among high school students. To address students’ sleep issues, it is essential to encourage the setting of appropriate goals while carefully considering each individual’s lifestyle.

### 6.3. The Importance of Sleep Improvement

The authors implemented a continuous sleep education program over a two-year period in collaboration with schools and families [32]. The outcomes of this program are described below. Through the sustained implementation of sleep-related lectures, parent education delivered via school health newsletters, and student-led activities conducted by the school health committee, improvements were observed not only in sleep-related lifestyle habits but also in psychosomatic health, motivation to improve sleep, and multiple aspects of school life.

The protocol of the current study was reviewed and approved by the Research Ethics Committee of Hiroshima International University (approval number 21-023, approval date 13 October 2021). The investigations were conducted in accordance with the principles outlined in the Declaration of Helsinki (1975, revised in 2013), and parental consent and student assent, together with consent from junior high school principals and class teachers, were obtained.

**Figure 2:**
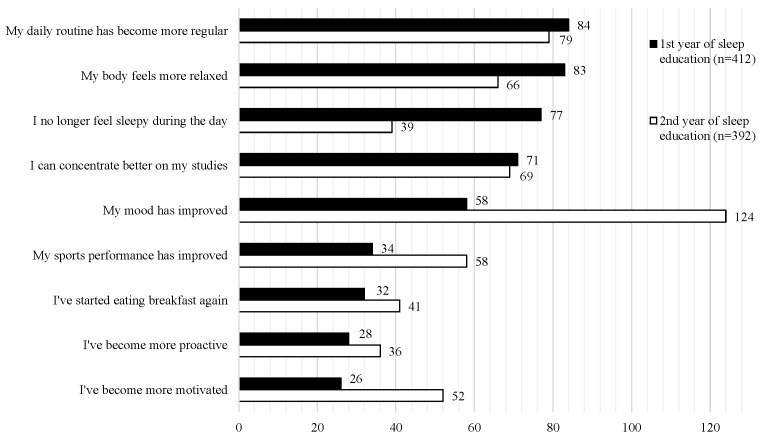
The effects of continuous sleep education on school life in junior high school [32].

Figure 2 summarizes the changes students felt in their school life due to improved sleep (multiple responses allowed) [32].

In the first year of sleep education implementation (black bars; n = 412), more than 80 students reported improvements in “their daily rhythm” and “how their body felt.” Additionally, more than 70 students reported “reduced daytime sleepiness” and “improved ability to concentrate on studies,” while more than 50 students reported feeling “more cheerful.” Interestingly, some students also noted “improved athletic performance,” “started eating breakfast,” “became more proactive,” and “felt more motivated.” At this school, a team of teachers, including school nurses and nutritionists, has continuously implemented these activities. Interestingly, in the second year (white bars in Figure 2; n = 392), the highest number of students (n = 124) reported feeling “more cheerful,” while the number of students reporting “improved athletic performance” and “increased motivation” also increased. Furthermore, among the 25 students whose social jet lag shifted from more than 1 h to less than 1 h between the first and second years, happiness scores increased. This suggests that ongoing sleep education may also be effective in improving students’ mental health and athletic performance.

## 7. Future Challenges for Sleep Education for Children and Adolescents

The implementation methods and evaluation approaches for sleep education and sleep management vary according to each school’s specific circumstances, but they broadly fall into the following three categories:

(1) Knowledge education and awareness-raising through a single lecture. In this case, a lecture (presentation) is given that incorporates true/false quizzes. The audience may consist of students only, or students together with parents and teachers.
(2) Knowledge education and Lifestyle Rhythm Healthy Method. In this case, a lecture incorporating true/false quizzes is provided, and goal setting is conducted using a lifestyle rhythm check.
(3) In addition to the above, participants record a sleep diary over a two-week period and engage in self-monitoring.

Effectiveness evaluation includes the following: (1) pre- and post-lecture true/false quiz accuracy rates and student feedback; (2) pre-lecture and two-week post-lecture assessments of knowledge acquisition (true/false quiz) and changes in habits and physical/mental condition. Meanwhile, the aforementioned lifestyle rhythm check and sleep log can also be applied to individualized guidance and sleep consultations.

The authors developed a one-page A4 sleep assessment form (Appendix B) to facilitate the efficient collection of data on children’s and students’ sleep issues and the evaluation of sleep education effectiveness in schools, and this tool is currently being utilized in practice [36]. This form includes questions about bedtime, wake-up time, and sleep duration, enabling the calculation of individual sleep debt, sleep deprivation, and the proportion of individuals going to bed after midnight. It also assesses levels of daytime impairment based on daytime sleepiness and irritability.

After assessing the situation, the tool can be used to set individualized goals based on each individual’s practice of sleep-promoting behaviors. In other words, this evaluation sheet is designed to be used both before and after sleep education to assess its effectiveness. Items 1–12 on the survey sheet are common to both elementary and junior high school students. By appropriately modifying the content of Item 13 according to the target group, the form can be widely applied.

If individuals are able to practice their target behaviors, establish healthier sleep habits, and experience tangible improvements in sleepiness, physical condition, and mood, they are more likely to continue these habits voluntarily. This questionnaire can therefore be utilized across a wide range of settings, from individual sleep consultations in the school health room to class-wide sleep education sessions. Children in Japan consistently fall short of the recommended sleep duration for their developmental age [2], with approximately one in four junior high school students and one in three high school students experiencing sleep deprivation. For the sake of children’s futures, sleep education must be addressed collaboratively by schools, families, and the broader community. In addition, other measures to protect students’ sleep, such as regulations on the start time of classes and working hours for minors, will also be necessary in the future.

## 8. Discussions

The objective of this review was to provide an overview of the effects of sleep education incorporating self-help treatment, a behavioral strategy, among Japanese children and adolescents, drawing on practical school-based examples. The results indicated that sleep education based on a short-term cognitive–behavioral approach, including sleep knowledge and self-help treatment, was associated with improvements in both sleep quality and duration, arousal levels, daytime concentration, and motivation. Self-help treatment appears to be effective in increasing the frequency of health-promoting behaviors, particularly when participants set their own target behaviors and self-regulate their implementation. Furthermore, the link between sleep knowledge and sleep-promoting behaviors plays a critical role in facilitating behavioral change [34,35]. Taken together, these findings suggest that both the acquisition of sleep knowledge and the practice of target behaviors may have contributed to improvements in sleep-promoting behaviors.

Previous four-week intervention programs targeting adolescents have been developed and implemented in Western countries and Hong Kong; however, most of these programs have failed to demonstrate sufficient effectiveness. One reason for this limited impact is that the proposed sleep-promoting behaviors were standardized rather than tailored to individual circumstances [25]. Another issue is that these interventions did not incorporate strategies to bridge the gap between sleep-related knowledge and actual engagement in sleep-promoting behaviors [37].

To address these challenges, the present sleep education package was designed not only to provide sleep-related knowledge and recommendations for sleep-promoting behaviors but also to incorporate a sleep-promoting behaviors checklist that enables students to assess their own implementation status. Based on this self-assessment, students set individualized goals and are encouraged to practice these target behaviors through structured methods. Previous studies have suggested that allowing participants to set their own goals and actively practice them is crucial for increasing the frequency of target behaviors [35]. Consistent with this notion, the study introduced in this review, which examined sleep-promoting behaviors among high school students, found that 57.3% of students showed improvements in their self-selected target behaviors during the post-intervention period.

Next, based on the probability that improvements in individually set target behaviors were associated with improvements in sleep duration, delayed bedtime, and sleep debt, we discuss which sleep-promoting behaviors were effective in improving these sleep-related problems. In this review, examination of sleep-promoting behaviors associated with improvements in sleep duration, bedtime, and sleep debt revealed that “waking up at approximately the same time every morning,” “keeping the difference between weekday and weekend wake-up times within two hours,” and “exposing oneself to sufficient sunlight after waking up in the morning” were associated with improvements in approximately 50–60% of students. These behaviors are key factors for synchronizing the circadian rhythm with the Earth’s 24 h cycle. Regular morning practice of these behaviors is known to advance circadian rhythm markers such as the dim light melatonin onset (DLMO) and sleep onset time [38,39].

Moreover, the time window during which light exposure advances the circadian phase is reported to occur approximately two hours after habitual wake-up time (e.g., around 8:30–9:00 a.m. for individuals who habitually wake at 7:00 a.m. on weekdays) [40]. Exposure to light after this phase-advance window does not advance the circadian phase and may instead induce a phase delay [11,12,13]. Thus, maintaining a consistent wake-up time every morning, ensuring exposure to sufficiently bright light, and limiting the difference between weekday and weekend wake-up times to within two hours may facilitate circadian phase advancement. These behaviors are therefore likely to be effective in advancing bedtime, which in turn may have contributed to improvements in sleep duration and reductions in sleep debt.

In addition, maintaining appropriate daytime arousal is essential for securing sufficient sleep duration. Behaviors important for sustaining arousal include “enjoying hobbies or club activities and spending the day actively” and “avoiding dozing off (napping) after returning home.” The present findings suggest that, among these behaviors, “not dozing off after returning home” was particularly important for improving sleep-related problems. It has been reported that 42.7% of Japanese high school students take naps lasting 30 min or longer between returning home from school and going to bed [41]. Evening naps are considered an indicator of sleep deprivation [42] and are closely associated with delayed bedtime and shortened sleep duration. Furthermore, because such naps contain a high proportion of delta waves, which reflect sleep pressure, they are known to make nocturnal sleep more shallow [43]. Considering these findings, refraining from dozing off during the evening and maintaining appropriate arousal levels may have been effective in preventing delayed bedtime and shortened sleep duration, thereby contributing to a reduction in sleep debt.

Furthermore, the findings indicated that avoiding the consumption of caffeine-containing beverages such as tea and coffee after dinner was important for improving sleep duration and bedtime. This is likely related to caffeine-induced hyperarousal. Previous studies have shown that high-dose caffeine intake close to bedtime can cause circadian phase delays, sleep fragmentation, and reduced sleep duration [44]. In recent years, high-caffeine beverages such as energy drinks have become increasingly prevalent, and a substantial proportion of high school students consume them habitually [45]. Therefore, students who reduced caffeine intake after dinner may have experienced decreased hyperarousal, which in turn may have contributed to improvements in bedtime and sleep duration. In addition, “going to bed by midnight” was also identified as an important behavior for improving sleep duration and bedtime. Given that shortened sleep duration is largely attributable to delayed bedtime, setting an earlier bedtime is particularly desirable on weekdays to ensure that students obtain the amount of sleep required by their bodies.

Taken together, the target behaviors that showed improvements are all sleep-promoting behaviors that play a critical role in alleviating common sleep problems among high school students, such as insufficient sleep duration, delayed bedtime, and sleep debt. These findings suggest that practicing individualized target behaviors may have fostered a positive cycle in which improvements in sleep problems lead to better daytime functioning, which in turn further supports healthy sleep.

Furthermore, sleep education has been continuously implemented over a two-year period in collaboration with school health teachers. Through sustained activities—including sleep lectures, awareness-raising initiatives for parents via school health newsletters, and student-led health committee activities—changes were observed not only in sleep-related lifestyle habits but also in physical and mental health, motivation for sleep improvement, and aspects of school life. These findings highlight the importance of collaboration between schools and communities in supporting children’s sleep and overall health. To promote and disseminate sleep education more broadly, it is essential to help students recognize that improvements in sleep can lead to positive changes in school life and to enable them to experience the tangible benefits of better sleep.

## 9. Conclusions

Sleep education programs incorporating self-help treatment not only enhance sleep-related knowledge but also promote sleep-promoting behaviors and improve sleep outcomes, thereby reducing daytime sleepiness among children and adolescents and supporting a healthy school life. The present findings suggest that sleep education based on a short-term cognitive–behavioral approach, including sleep knowledge and self-help treatment, is associated with improvements in sleep quality, arousal levels, daytime concentration, and motivation. Based on these findings, we emphasize the importance of sleep education and sleep management within school settings and propose that effective sleep improvement support requires (1) the dissemination of appropriate knowledge, (2) the provision of practical support tools, and (3) the development of trained human resources.

In Japan, there has traditionally been a strong emphasis on perseverance, endurance, and working without adequate rest, which has contributed to the historical undervaluation of sleep. However, to support the physical and mental health, healthy development, and optimal functioning of children and adolescents, it is necessary to re-examine the importance of sleep. Moreover, improving sleep requires not only knowledge about sleep health but also strategies that effectively translate such knowledge into sleep-promoting behaviors. To facilitate changes in both awareness and behavior, it is important to encourage individuals to reflect on their own lifestyles and to set appropriate, personalized goals. In addition, understanding and support from family members and teachers play a crucial role in fostering sustainable improvements in sleep.

## Figures and Tables

**Table 1:** The sleep-promoting behaviors list for junior high school students.

Sleep-Promoting Behaviors	Self- Assessment		
1. Getting up at almost fixed-time every morning	〇	△	×
2. Exposing yourself to sunlight in the morning	〇	△	×
3. Having breakfast every morning	〇	△	×
4. Not taking a nap just after coming back home	〇	△	×
5. Avoiding to have caffeinated drinks, such as tea or coffee, after dinner	〇	△	×
6. Avoiding to have snacks late in the evening	〇	△	×
7. Taking a tepid bath relaxedly by 2 hrs before bedtime	〇	△	×
8. Going to bed by 12:00 am at the least every night	〇	△	×
9. Trying to rest the brain and mind before sleep	〇	△	×
10. Keeping wake-up time on weekends not more than two hrs later than that on weekdays	〇	△	×

“〇 practice”, “× do not practice”, and “△ but can practice”.

**Table 2:** Changes in target behaviors with sleep education [36].

				Pre			Post										*p*
				Target Behavior			Improved			No Change			Worsened			McNemar χ2	
				n	(%)		n	(%)		n	(%)		n	(%)			
All sleep-promoting behaviors				478	(100)		274	(57.3)		186	(38.9)		4	(0.8)		260.29	<0.001
1. Getting up at a fixed time every morning				43	(9.0)		26	(60.5)		12	(27.9)		0	(0)		24.04	<0.001
2. Exposure to sunlight in the morning				86	(18.0)		33	(38.4)		51	(59.3)		2	(2.3)		25.71	<0.001
3. Having breakfast every morning				11	(2.3)		6	(54.5)		4	(36.4)		1	(9.1)		4.17	0.031
4. Enjoying the club activities and hobbies during the daytime				20	(4.2)		17	(85.0)		2	(10.0)		0	(0)		15.06	<0.001
5. Avoiding taking a nap immediately after returning home from school				51	(10.7)		25	(49.0)		25	(49.0)		0	(0)		23.04	<0.001
6. Avoiding caffeinated behaviors, such as coffee or tea, after dinner				41	(8.6)		21	(51.2)		20	(48.8)		0	(0)		19.05	<0.001
7. Not going out to brightly lit places after 9:00 pm				16	(3.3)		13	(81.3)		1	(6.3)		0	(0)		11.08	<0.001
8. Keeping a regular bedtime every day				53	(11.1)		33	(62.3)		17	(32.1)		2	(3.8)		25.71	<0.001
9. Going to bed by 12:00 am at the latest every night				44	(9.2)		26	(59.1)		16	(36.4)		0	(0)		24.04	<0.001
10. Avoiding worries while in the bed				24	(5.0)		16	(66.7)		8	(33.3)		0	(0)		14.06	<0.001
11. Keeping wake-up time on weekends within 2 h of that on school days				50	(10.5)		34	(68.0)		15	(30.0)		0	(0)		32.03	<0.001
12. Keeping a regular sleep time every day				39	(8.2)		24	(61.5)		15	(38.5)		0	(0)		22.04	<0.001

*Note*. Improved = Pre: “△ but can practice” → Post: “〇 practice”, no change = Pre: “△ but can practice” → Post: △, “do not practice”, worsend = Pre: “△ but can practice” → Post: “× do not practice”.

**Table 3:** Probability of improvement in sleep duration, bedtime, and sleep debt when target behave ior is improved.

				Increase Sleep Time (40 min ≧)	Advance Bedtime (30 min)	Reduce Sleep Debt (60 min)
			
			
1. Getting up at a fixed time every morning				65.3	73.8	73.8
2. Exposure to sunlight in the morning				58.3	56.4	66.7
3. Having breakfast every morning				27.6	48.8	80.0
4. Enjoying the club activities and hobbies during the daytime				18.2	25.0	25.0
5. Avoiding taking a nap immediately after returning home from school				77.6	74.3	87.4
6. Avoiding caffeinated behaviors, such as coffee or tea, after dinner				81.2	81.2	59.6
7. Not going out to brightly lit places after 9:00 pm				31.8	37.5	50.0
8. Keeping a regular bedtime every day				43.9	50.6	55.7
9. Going to bed by 12:00 am at the latest every night				84.2	82.1	55.9
10. Avoiding worries while in the bed				42.9	20.0	71.4
11. Keeping wake-up time on weekends within 2 h of that on school days				77.1	84.0	85.7
12. Keeping a regular sleep time every day				67.6	42.2	27.3

*Note*. The numbers in the table represent probability values (%).

**Figure A1:**
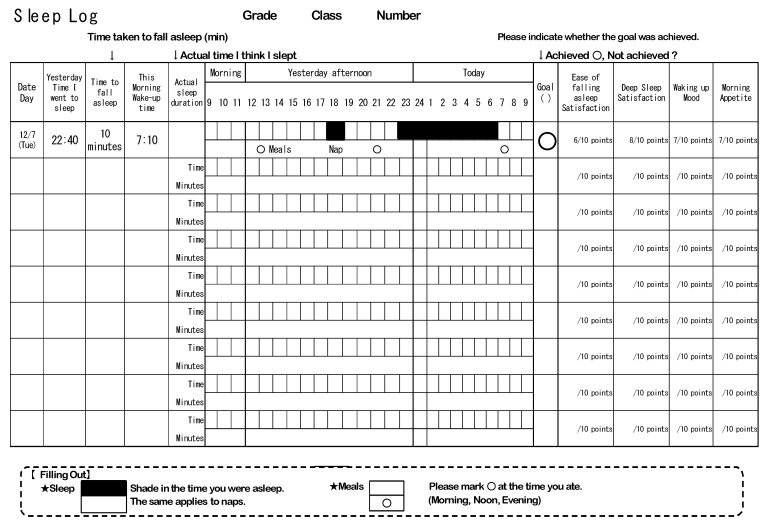
Sleep log for the sleep education program with self-help treatment.

**Figure A2:**
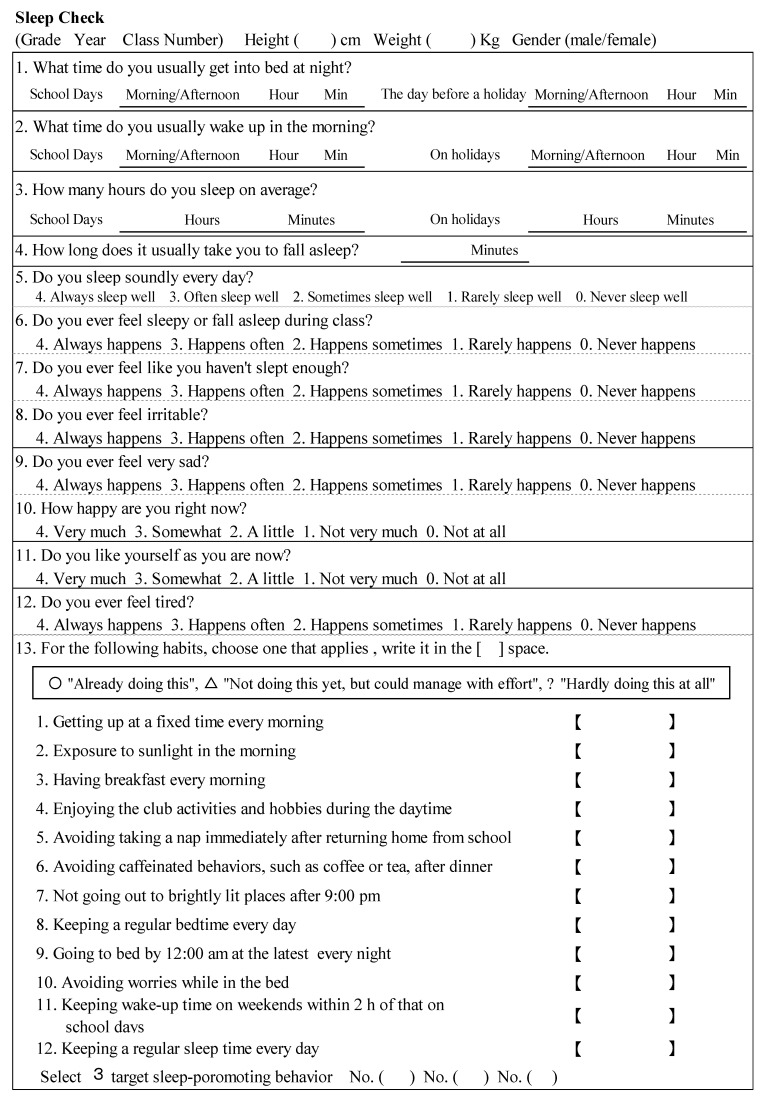
Sleep Checklist (a one-page A4 sleep assessment for sleep education).

## Data Availability

No new data were created or analyzed in this study.
